# F173. PITCH AND DURATION MISMATCH NEGATIVITY, AUDITORY CORTEX GRAY MATTER, AND PRODROMAL ROLE FUNCTIONING IN THE FIRST EPISODE SCHIZOPHRENIA SPECTRUM

**DOI:** 10.1093/schbul/sby017.704

**Published:** 2018-04-01

**Authors:** Dean Salisbury, Anna Shafer, Brian Coffman, Timothy Murphy

**Affiliations:** 1 University of Pittsburgh School of Medicine; 2 University of Pittsburgh

## Abstract

**Background:**

Primary auditory cortex, contained within Heschl’s gyrus, is implicated auditory processing deficits and auditory verbal hallucinations in schizophrenia. Previously we showed a pathological correlation between the magnitude of the pitch-deviant mismatch negativity (pMMN) response during a passive auditory task and reductions in gray matter volume in Heschl’s gyrus in subjects with first hospitalized for schizophrenia. The aim of this study was to replicate this finding, examine duration-deviant mismatch negativity (dMMN) and gray matter correlations, and to examine pre-psychosis role functioning, in a first episode psychosis sample within the schizophrenia-spectrum.

**Methods:**

Participants included 40 first episode schizophrenia subjects (FESz) and 40 healthy controls (HC) matched for age, parental socioeconomic status, IQ, sex, and handedness. For MMN extracted from the EEG, standard tones were presented repeatedly (1 kHz, 75 dB, 50 ms pips, 5 ms rise/fall times, 330 ms SOA) with an occasional pitch deviant (1.2 kHz, 10% of trials) or duration deviant (100 ms, 10% of trials) interspersed. pMMN and dMMN were measured from subtraction waveforms as the average voltage within a 100-ms group averaged peak window at Fz. Role functioning was measured with the Cornblatt Global Functioning: Role scale. A subset of 28 FESz and 28 matched HC underwent structural MRI. High-resolution T1-weighted structural MRI data (3T) were acquired for each subject. Freesurfer was used to segment white matter, gray matter, and pial surfaces. Left and right Heschl’s gyri were manually edited regions of interest, and gray matter volumes determined.

**Results:**

Despite a lack of pMMN or dMMN reduction at the group level in FESz, both measures were pathologically correlated with role functioning in the year prior to hospitalization. In FESz, smaller pMMN at Fz was associated with poorer role functioning in the year prior to psychosis (rho= -.35, p =.03). Similar associations were observed for dMMN (rho= -.41, p <.01). Furthermore, in the subset of FESz with sMRI, smaller pMMN at Fz was associated with less total gray matter volume in left Heschl’s gyrus (TGMV) (rho= -.40, p =.03) but not right. Similar associations were observed for dMMN (rho= -.47, p .01). As well, role functioning and auditory cortex gray matter volumes were not correlated in FESz. There were no significant correlations within HC.

**Discussion:**

Although pMMN and dMMN are not reduced at the group level, the size of both are associated with impaired functioning prior to psychosis and reduced gray matter volume of left hemisphere Heschl’s gyrus, containing primary and secondary auditory cortices. Thus, pMMN and dMMN although not sufficient as biomarkers of disease presence, are suitable as reliable biomarkers of disease progression. Presumably, poorer role functioning and less gray matter reflect more of the pre-psychosis progressive pathological process thought to occur in the prodromal phase of psychosis. Hence, pMMN and dMMN are likely to serve as sensitive and robust outcome measures for therapeutic interventions and to guide treatment strategies in the prodrome and during early psychosis.

